# Menadione degrades the optical quality and mitochondrial integrity of bovine crystalline lenses

**Published:** 2011-01-26

**Authors:** Kenneth W. Olsen, Vladimir Bantseev, Vivan Choh

**Affiliations:** 1 Institute of Medical Science, University of Toronto, Toronto, ON, Canada, and Department of Ophthalmology and Vision Sciences, Toronto Western Hospital, Toronto, ON Canada; 2 Genentech Inc, South San Francisco, CA; 3 School of Optometry, University of Waterloo, Waterloo, ON, Canada

## Abstract

**Purpose:**

The crystalline lens is a unique cellular organ that performs metabolic processes while maintaining transparency for optical functionality. Mitochondria play a role in providing cells with aerobic respiration necessary for these metabolic processes. Using menadione, a mitochondria-specific inhibitor of the quinone family, and bovine lenses in vitro, this study was undertaken to determine whether a relationship exists between mitochondrial function and optical function.

**Methods:**

Bovine lenses were treated with 50 μM, 200 μM, 600 μM, and 1,000 μM menadione and lens optical function, assessed as optical quality, was observed over 9 days. Confocal micrographs of mitochondria in superficial secondary fiber cells were also analyzed in 50 μM, 200 μM, and 600 μM menadione-treated lenses over 48 h.

**Results:**

A decrease in lens optical quality was observed in a dose-dependent manner within 24 h for the 200 µM- (p=0.0422), 600 µM- (p<0.0001), and 1,000 μM- (p<0.0001) treated lenses. No change in optical quality was observed for the 50 μM-treated lenses. Analysis of confocal micrographs indicated a trend of shorter mitochondria for 200 μM- and 600 µM-treated lenses with time and analysis of the distributions of mitochondrial lengths indicated a relative increase in the number of shorter mitochondria with higher doses of, and longer exposures to, menadione.

**Conclusions:**

The data show that menadione has a detrimental effect on mitochondrial integrity and this change is associated with degradation of optical quality, suggesting a possible link between mitochondrial function and optical function.

## Introduction

As a living and constantly growing cellular organ, the crystalline lens must carry out the usual ATP-essential metabolic processes required for maintenance and growth, while also ensuring transparency to allow for the proper optical functionality of fine focusing light onto the retina. While originally thought to be absent from superficial fiber cells of crystalline lenses, and few in number within lens epithelial cells, mitochondria have recently been shown to be more numerous [1] and more dynamically active [2] in both cell types than was previously thought. These new findings suggest that mitochondria may contribute more to overall lens metabolism than was once believed.

Within the bovine crystalline lens, mitochondria account for approximately 33% of all ATP produced through oxidative phosphorylation, while the remaining ATP present results from glycolysis [3]. Despite the need for ATP production, mitochondria pose a potential problem to transparency, since in a structure as thick as the lens, mitochondria could scatter light [4]. To maintain lens transparency, a part of secondary lens fiber differentiation includes the degradation of mitochondria (as well as other membrane bound organelles) within and in areas just adjacent to the lens nucleus [5], called the organelle-free zone. Thus, mitochondria in the lens are localized to the anterior epithelium and to the most superficial fiber cells. The activity of the remaining mitochondria appears to be important for normal functioning of the lens, as disruption of the natural organelle degradation process during development by premature inactivation of the mitochondrial oxidative phosphorylation of superficial mitochondria causes the organelle-free zone to develop opacities, known as cataracts [6,7]. Given these findings, the mitochondria of the superficial cortex must play a great role in lens metabolism and possibly cataract formation, even though they occupy only a minute portion of the lens.

The purpose of this study was to evaluate the relationship between mitochondrial function (assessed by mitochondrial integrity) and optical function of the bovine crystalline lens using the mitochondrial uncoupler menodione at different concentrations to understand mechanisms of toxicity and possible recovery from this model chemical. Menadione (2-methyl-1,4-naphthoquinone), also known as vitamin K_3_, is a member of the quinone family and is known to have both toxic [8] and non-toxic effects [9]. Toxic effects occur principally by a one-electron reduction in mitochondria. At high levels, menadione is detrimental to cells [10,11], however, at low levels, menadione has been shown to be non-toxic and may even improve mitochondrial function [12]. This study was undertaken to examine how the integrity of the mitochondria affects lens transparency. As no studies to date have been performed on cultured bovine lenses using menadione, the effectiveness of menadione as a bovine lens mitochondrial uncoupler was also verified.

## Methods

### Eye dissection

Whole bovine eyes were obtained from a local abattoir (Cargill Meat Solutions Ltd., Guelph, ON, Canada). Using aseptic techniques, eyes were dissected and lenses were carefully excised, then immediately placed on a plastic ring suspended in a glass chamber containing 23 ml of culture medium M199. Culture medium consisted of M199 (M3769; Sigma, St. Louis, MO) modified with 26.2 mM sodium bicarbonate, 25.0 mM 4-(2-Hydroxyethyl)piperazine-1-ethanesulfonic acid (HEPES), 0.68 mM L-glutamine, 0.7% sodium hydroxide, 1% penicillin/streptomycin, and 3% qualified fetal bovine serum. During the study, lenses were incubated at 37 °C with 5% CO_2_ and replaced with fresh culture medium every 48 h. Following 24 h of incubation lenses were visually inspected and those showing signs of mechanical damage were discarded.

### Lens treatment

Using aseptic techniques, the lenses were exposed for 30 min to culture medium containing 0 μM (control, n=10), 50 μM (n=9), 200 μM (n=9), 600 μM (n=8), and 1,000 μM (n=9) of menadione salt (M2518; Sigma, Oakville, ON) dissolved in culture medium at room temperature. Lenses were then washed three times with 0.9% saline solution, once in serum-free M199 and then returned to their chambers containing fresh M199. Lenses used as control (n=10) were treated with medium and washed in the same manner.

### Assessment of optical function

Lens optical quality and sharpness of focus were assessed using the Scantox™ In vitro Lens Assay System (XTOX Scientific, Napean, ON) [1], which captures images of refracted laser beams passing through the lens at various eccentricities from the optical axis. The distance between the most posterior point of the lens (the back vertex of the lens) and the point at which the refracted beam crosses the optical axis (focal point) is the back vertex distance (BVD). In this study, lenses were optically scanned before treatment, then again at 4, 24, 48, 144, and 216 h post treatment, using 22 laser beams per lens at each time point. BVD variability, calculated as standard error, was used as an indicator of optical quality, with greater BVD variability indicating worse optics. Only lenses with pre-treatment (baseline) BVD variability values between 0.200 and 0.400 mm were included in this study.

### Assessment of secondary fiber cell mitochondrial integrity

For mitochondrial integrity analysis, lenses were carefully removed from their chambers and incubated in 20 µM Rhodamine 123 (R-302; Molecular Probes, Eugene, OR) in serum-free M199 for 20 min at 37 °C. The lenses were then rinsed in serum-free medium. Each lens was placed on its equatorial region and immobilised on a Corning No. 1 (VWR, Mississauga, ON) cover glass glued to the bottom of a well (6-well plate) using 1% (w/v) agarose. The agarose was previously melted in M199 and cooled to 35 °C.

Mitochondrial integrity in secondary fiber cells was assessed using a Zeiss confocal laser scanning microscope (CLSM; Carl Zeiss, Toronto, ON) 510 META system connected to an Axiovert 200 inverted microscope (Carl Zeiss) equipped with a water immersion C-Apochromat (color corrected) 40× objective (NA 1.2). Rhodamine 123 fluorescence was visualized using an argon/krypton laser with a 514 nm excitation laser line and a 560 nm long-pass emission filter. Micrographs of secondary fiber cells residing in the superficial cortex were taken for 0 µM (control), 50 µM, 200 µM, and 600 µM menadione-treated lenses at 4, 24, and 48 h post treatment. Since the Scantox™ results indicated that most of the damage had occurred by 48 h, mitochondrial integrity was assessed up to that time point. Similarly, the results for the 1,000 µM lenses were not different from those of the 600 µM lenses and therefore 1,000 µM lenses were not assessed.

Mitochondria were counted and their average and total lengths were measured in micrographs of secondary fiber cells using analysis software script written for MATLAB (Mathworks, Natick, MA). Micrographs from three areas per lens were used in the analysis with three lenses per treatment group per time point, for a total of 108 micrographs analyzed. For each micrograph, analysis consisted of retracing each individual mitochondrion using both an automated process and the manual use of the fine adjustment tools of the analysis software to ensure that mitochondria information was correctly calculated. To examine how the distributions of mitochondrial lengths and numbers changed as a function of exposure time to and concentration of menadione, frequency histograms of mitochondrial lengths were generated for all mitochondria within each lens. As the longest observed mitocondrion was 112.29 μm in length, histogram plots with 57 bins of 2 μm increments were used.

### Statistical analysis

BVD variability data were normalized using a log transformation to account for inhomogeneous variance between the dosage groups. Data were analyzed using a two-way repeated measures analysis of variance (ANOVA) with Tukey or Bonferroni post-hoc tests. Greenhouse-Geisser corrections were used for epsilon values of less than 0.75. Mitochondrial lengths, mitochondrial numbers and mitochondrial distributions were analyzed using two-way ANOVA tests with Tukey post-hoc tests where necessary. For all tests, results were considered significant at p≤0.05.

## Results

### Optical function

**Figure 1:**
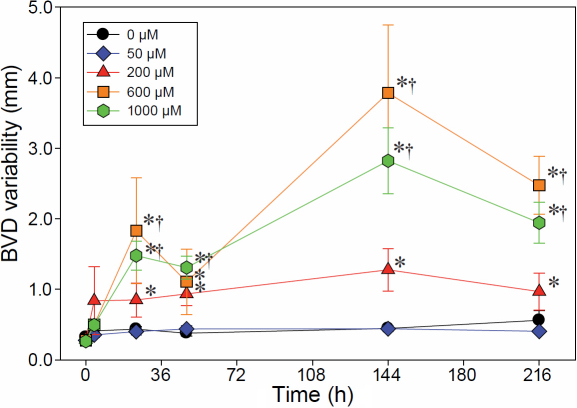
Effects of menadione on back vertex distance variability. Line graphs show the effects of 0 µM (control; black circles), 50 μM (blue diamonds), 200 μM (red triangles), 600 µM (orange squares) and 1,000 µM (green hexagons) menadione treatment on BVD variability (±standard error). Asterisks (*) denote significant changes (p≤0.05) in BVD variability with respect to its pre-treated scan (time 0). Daggers (†) denote significant changes in BVD variability with respect to the control group (0 µM) at the given time point. Results show an increase in BVD variability as a function of menadione concentration and time.

Treatment of bovine lenses with menadione in vitro was associated with both a time-dependent (p<0.0001) and a concentration-dependent (p<0.0001) increase in BVD variability, indicating a decrease in optical quality (Figure 1). Interactions were also noted between the two factors (time and concentration; p<0.0001). No changes in BVD variability were observed within the 0 μM- (control) and 50-μM treated lenses over time (p=0.6200 and p=0.1130, respectively) but for all other dosage groups, differences in BVD variability changed as a function of time. BVD variability in the 200-μM treated lenses showed increases relative to baseline (time point 0) beginning at 4 h, but changes were not significant until 24 h after treatment (p=0.0422). The increased BVD variability for this group was maintained through the 48, 144, and 216 h time points (p=0.0002; p<0.0001; p=0.0025, respectively). Lenses treated with 600 μM menadione also showed significantly higher BVD variability at all time points relative to the baseline beginning at 24 h (24, 48, 144, and 216 h; p<0.0015 for all time points). In addition, these lenses showed two time points of peak BVD variability, the first, smaller increase at 24 h (p<0.0001), and a second, larger increase at 144 h (p<0.0001), with both BVD variabilities at these two time points also significantly different (p=0.0424) from each other. The decrease in BVD variability at 48 h separating the two peak values was significantly different from the second peak (144 h: p<0.0001) but not the first (p=1.000). The 1,000 µM-treated lenses responded in a similar manner as those for the 600 µM group, with significant increases at 24, 48, 144, and 216 h (p<0.0001 for all time points) and apparent peak BVD variabilities at 24 and 144 h; however, for this group, the second peak BVD variability at 144 h was not different from the earlier peak at 24 h (p=1.0000) and the decrease between the peaks was also not different from either peak value (p=1.000 and p=0.3076, respectively).

As might be expected given the time course of differing menadione doses, there were differences between the drug effects at various time points (p<0.0001; Figure 1). No dosage effects were detected over the first 4 h (p=0.6056), but beginning at 24 h, BVD variability for two highest dosage groups were significantly different than those for the two lowest dosage groups. Specifically, BVD variability in the 600 µM and 1,000 µM groups were greater than those for the 0 µM group (p=0.0030 and p=0.0010, respectively) and 50 µM group (p=0.0013 and p=0.0005, respectively). The BVD variability for the 200 µM-treated lenses was intermediate to the low and high dosage level groups and showed no significant differences to any of the other groups (0 µM: p=0.5949; 50 µM: p=0.3815; 600 µM: 0.1205; 1,000 µM: p=0.0585). At 48 h, BVD variability of the 1,000 µM was still significantly higher than those of the 0 µM and 50 µM groups (p=0.0001 and p=0.0043, respectively) but BVD variability for the 600 µM group was no longer different from those of the two lowest dose groups (p=0.3941 and p=0.8950). The second peak value (at 144 h) showed much the same pattern as for the first, with the 600 µM- and 1,000 µM-treated lenses showing significantly greater BVD variabilities than those of the 0 µM- and 50 µM-treated lenses (p<0.0001 for all comparisons). However, for this time point, the BVD variability for the 200 µM-treated lenses was also different from those for the 600 µM and 1,000 µM groups (p=0.0026 and p=0.0300, respectively), but not different from those for the 0 µM and 50 µM groups (p=0.2051 and p=0.2268, respectively). This pattern was mostly maintained for the last time point (216 h), with the sole exception of the 200 µM-treated lenses showing similar BVD variability to that of the 1,000 µM-treated lenses (p=0.0601).

### Secondary fiber cell mitochondrial integrity

**Figure 2:**
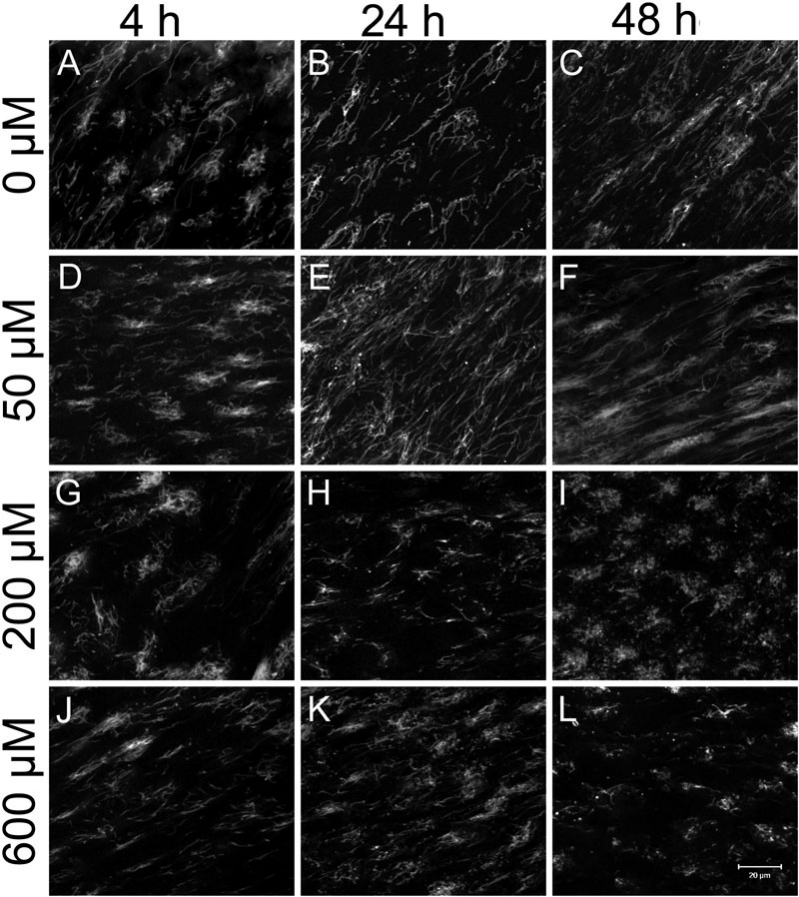
Effects of menadione on mitochondrial integrity.  Representative confocal micrographs of mitochondria in secondary fiber cells for 0 µM (**A**-**C**), 50 µM (**D**-**F**), 200 µM (**G**-**I**) and 600 µM (**J**-**L**) menadione-treated lenses at 4 h, 24 h, and 48 h.  Scale bar represents 20 µm.  Note the increase in the number of smaller mitochondria as menadione concentration and time increase.

Mitochondrial integrity was assessed using confocal images of lens fiber cells, with shorter mitochondrial lengths and/or increased numbers of mitochondria taken to indicate more damaged mitochondria. Visual assessment of the confocal images showed that in general, shorter mitochondria and greater numbers of mitochondria were associated with increasing concentrations of, and exposure times to, menadione (Figure 2), indicating overall damage to the mitochondria. The sum of the lengths (total length) of all mitochondria was taken to represent the total amount of mitochondria (Table 1). No changes as a function of time of exposure to (p=0.5837) nor concentration of (p=0.1090) menadione was detected; nor was any interaction detected between the two factors (p=0.3236), which suggests no changes in the total amount of mitochondrial material. However, the average number of mitochondria per lens did increase, both as a function of concentration (p=0.0488) and of time (p=0.0263), and there was significant interaction between the two factors (p=0.0059; Table 1). Overall, the average number of mitochondria in 600 µM-treated lenses was significantly higher than that in the 0 µM-treated group (p=0.0456). The greatest change in mitochondrial numbers occurred in the 200 µM group; mitochondrial numbers at 48 h were significantly greater than those at 4 h and 24 h (p=0.0027 and p=0.0096, respectively). Also at 48 h, the number of mitochondria for the 200 µM group was significantly greater than those at the same time points for the 0 μM (p=0.0217) and 50 μM (p=0.0306) groups. In contrast, analysis of the number of mitochondria per micrograph showed no differences at the 4 and 24 h time points (p>0.4415 for all time points) for all dosage groups.

**Table 1:** Average length of mitochondria, the number of mitochondria (count), and total length of mitochondria from confocal microscopy micrographs.

** **	**Average length±s.d. (µm)**			**Number±s.d.**			**Total length±s.d. (µm)**		
**Concentration of menadione**	**4 h**	**24 h**	**48 h**	**4 h**	**24 h**	**48 h**	**4 h**	**24 h**	**48 h**
0 µM	7.6±1.3	7.1±1.7	7.7±3.3	244±10	329±29	247±57	1848±275	2322±464	1814±788
50 µM	8.7±2.2	8.2±2.7	8.2±2.9	248±27	379±105	260±33	2205±799	3229±1610	2051±636
200 µM	7.9±0.8	6.4±2.6	3.7±0.9	165±57	214±73	607±190*†	1286±416	1414±767	2114±635
600 µM	5.9±1.0	5.1±2.6	3.6±0.6	380±36	431±286	447±52	2232±469	1730±781	1618±451

Asterisk (*) denotes significant changes (p≤0.05) with respect to its 4 h time point. Dagger (†) denotes significant change with respect to 0 μM-treated lenses at the given time point. A trend toward a shorter average length and an increased number of mitochondria is observed.

**Figure 3:**
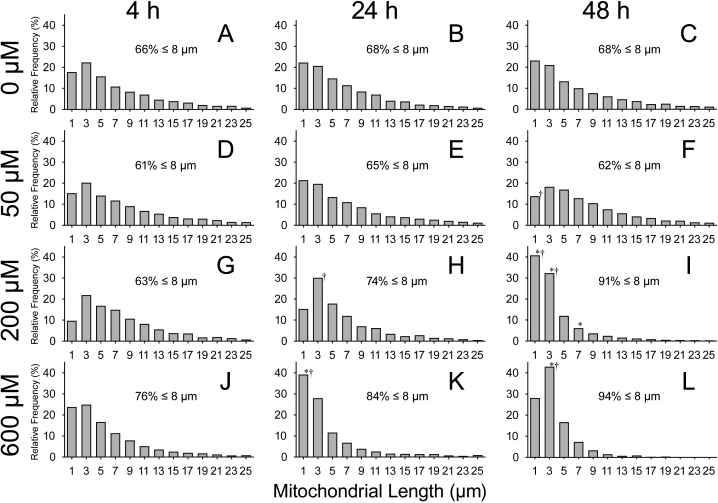
Relative distributions of mitochondrial lengths.  Histogram plots show the relative distributions of mitochondrial length for 0 µM (**A**-**C**), 50 μM (**D**-**F**), 200 µM (**G**-**I**), and 600 µM (**J**-**L**) menadione-treated lenses at 4 h, 24 h, and 48 h.  Only mitochondria less than 26 µm in length, comprising a minimum of 95% of the total number of mitochondria, are shown.  Percentages indicating the cumulative relative frequency of mitochondria ≤8 µm in length are also shown on each graph.  Asterisks (*) denote significant change over time within a given concentration treatment group with respect to the 4 h time point.  Daggers (†) denote a significant change in relative distribution for a given mitochondrial size with respect to 0 µM-treated lenses.  A general shift toward a greater relative frequency of smaller mitochondria as a function of both time and concentration is observed.

Despite the increase in mitochondrial number as a function of both dose of and exposure to menadione, statistical tests could only verify an overall concentration-dependent difference (p=0.0082) in mitochondrial lengths between the 50 μM and 600 μM groups (p=0.0087), with those of the 50 μM-treated lenses being longer (Table 1). No difference over time (p=0.1417) nor any interaction between exposure time and concentration factors could be detected (p=0.6472). However, the trends for the mitochondrial lengths for the 200 µM and 600 µM groups to almost halve by 48 h suggested that there were subtle changes to the lengths that could not be analyzed with means alone. Relative frequency distribution histograms were generated for each lens to characterize the overall distribution of the mitochondria based on length. For presentation purposes, histogram plots showing lengths only up to 26 µm, comprising at least 95% of the total number of mitochondria, are shown (Figure 3). All histograms were skewed to the right, indicating a preponderance of shorter mitochondria for all lenses. However, shifts in the distribution to the left, indicating an increase in the number of smaller mitochondria, a disappearance of larger ones, or a combination of the two, were observed as the concentration of menadione or exposure time to menadione increased (compare Figure 3A to Figure 3L). This observation is also borne out by assessments of the cumulative frequencies of the histograms; in general, the proportion of mitochondria that are shorter than 8 µm in length tends to increase as the concentration and exposure time menadione increases (c.f.: 66% versus 94%).

Analysis of the relative frequency distribution histograms indicated changes in the numbers of the smaller mitochondria depending on post-treatment time and concentration (p<0.0001). As expected, no change in distribution was detected for the 0 μM or 50 μM treatment groups over time. The 200 μM-treated lenses contained more short mitochondria for the 48 h (0–2 µm: p=0.0002, 2–4 µm: p=0.0002, 6–8 µm: p=0.0183) time point when compared with the 4 h time point. Lenses treated with 600 µM menadione showed significantly increased numbers of shorter mitochondria at both the 24 h (0–2 µm: p=0.0002) and 48 h (2–4 µm: p=0.0002) time points compared to the 4 h time point. When compared to the 0 µM-treated lenses, a relative increase in shorter mitochondria was observed for the 200 μM- (2–4 µm: p=0.0010) and 600 μM- (0–2 µm: p=0.0002) treated lenses at the 24 h time point. A similar increase in shorter mitochondria was found at the 48 h time point for 200 μM- (0–2 µm and 2–4 µm: both p=0.0002) and 600 μM- (2–4 µm: p=0.0002) treated lenses compared to the 0 μM treatment group. In contrast, the 50-μM treated lenses showed a decrease in relative distribution for the mitochondria shorter than 2 µm in length (p=0.0074) at the 48 h time point compared with the 0 μM treatment group, indicating a shift toward a higher relative distribution of longer mitochondria.

## Discussion

We used a scanning laser monitor (ScanTox™) to assess how menadione-induced changes to mitochondria might affect the optical quality of lenses in vitro and have found that menadione has effects at lower concentrations and in shorter exposure times than previously reported. Hedge and Varma [13] reported cataractogenesis in rat lenses that were exposed to 1,000 µM menadione for 5 h, while we found signficant optical degradation in 200 µM-treated lenses by 24 h. The increased sensitivity of a lenticular in vitro model used with a scanning laser monitor suggests a more sensitive means of objectively determining toxicity effects. Confocal microscopy provides high resolution and greater contrast images superior to conventional light microscopy, but without the artifacts associated with electron microscopy [14,15]. Together with the scanning laser monitor, we have in our possession a powerful means to record important toxicity parameters previously difficult to measure using other in vitro methods [16].

The results showing that the optical quality of lenses exposed to increasing concentrations of menadione becomes increasingly degraded (Figure 1) indicates an association between optical quality and health of mitochondria within the lens. The toxic effects of menadione on optical degradation seem to be grouped in a linear manner, with 0 µM and 50 µM treatments producing no toxic effects, the 600 μM and 1,000 µM treatments resulting in significant degradation and the 200 µM treatments generating changes intermediate to the lower and higher concentration treatment groups (Figure 1). The lack of optical differences between 600 μM- and 1,000 μM-treated lenses were taken to indicate that a maximal concentration, at which menadione effects were saturated, had been reached. As a result, increasing concentrations beyond this saturation point would have no additional detectable toxic effects.

It has been previously demonstrated that the optical quality of a lens can recover from mild toxic exposure [1,17,18]. The observations of decreases or “dips” in the BVD variability between at 24 h and 48 h in lenses exposed to the two highest concentrations (600 µM and 1,000 µM; Figure 1) might suggest at least a partial recovery from menadione effects, where the lens begins to overcome the initial toxic insult before being overwhelmed by the toxin. This idea raises the question of why the 200 µM-treated lenses showed no recovery. However, examination of the 200 µM-treated lenses indicates that recovery effects did occur in these lenses, albeit not in all of them (data not shown); 5 out of the 9 lenses showed recovery, leading to an overall difference in optical quality compared to the 600 µM (p=0.0055) and 1,000 µM (p=0.0488) treatment groups (data not shown). These findings may suggest that there is a certain threshold of damage that the lens will tolerate and recover from before being overwhelmed.

While the first decrease in BVD variability for the 600 µM- and 1,000 µM-treated lenses might be acceptable as a partial recovery point, it is highly unlikely that the second decrease in BVD variability (at 216 h) observed for these lenses could be a recovery point. First, analysis of the mitochondrial integrity suggests that the mitochondrial damage would be too great at this time point for recovery to occur; by 48 h (the last time point for which confocal images of mitochondria were collected), some areas of 600 µM-treated lenses already contained no mitochondria, indicating complete mitochondrial degradation (data not shown). Second, it was observed that as lenses became more damaged, more beams were scattered beyond the range of the cameras, resulting in fewer of the refracted laser beams captured by the cameras (data not shown). As a result, those longer focal lengths that would have contributed to a higher BVD variability were not included in the calculation, resulting in falsely lower values for BVD variability. Thus, the more damaged a lens becomes, after a certain point, the better the BVD variability would appear, since the most scattered laser beams would be excluded from the analysis. This calculation artifact is a limit of the optical scanner; artificially lower BVD variabilities can arise when lenses are extremely damaged so that scatter is too great and the few “good” beams that can be captured, are used for the variability calculations.

Given that no changes were detected in the total amount of mitochondrial material, but that mitochondrial numbers increased with increased exposure to and concentrations of menadione (Table 1), changes in mitochondrial lengths were also expected but were not evident with analysis of means alone (Table 1), most likely a result of low power (the micrograph analysis is highly labor intenstive and analysis of 108 micrographs is equivalent to only n=3). However, the distributions of the various-sized mitochondria indicate that the effects on 200 μM-treated lenses again lie intermediate to the those exposed to higher dosed lenses (600 µM) and lower dosed (0 µM and 50 µM) lenses; at 4 h post-treatment, average lengths are similar to those of 0 μM- and 50 μM-treated lenses at the same time point, but by 48 h, average lengths resemble those of the 600 μM-treated lenses. These data indicate that, as with optical degradation in the lens, increasing doses of menadione were generally associated with a linearly increasing degradation of mitochondria. Moreover, the time course for changes in optical quality and in mitochondrial integrity both center around 24 h, suggesting an association between mitochondria integrity and morphology with the optic functionality of the lens. The finding that mitochondria in the 600 μM-treated lenses are generally shorter across all time points compared to those for the other three treatment groups is also consistent with the idea that fragmented mitochondria in the lens is associated with a loss of optical quality.

A link between increasing BVD variability and loss of mitochondrial integrity has been previously shown by Bantseev et al. [1] using carbonyl cyanide m-chlorophenylhydrazone (CCCP), a mitochondrial uncoupler that is known to cause mitochondria to fragment [19,20]. That mitochondrial integrity is adversely affected by menadione, resulting in an increase in the number of smaller sized mitochondria, suggests that menadione too, might also act as a mitochondrial uncoupler in the vertebrate lens. A recent study by Makino et al. [21] showed that mitochondria in diabetic mouse coronary endothelial cells (MCEC) fragmented in response to menadione exposure. Menadione produces reactive oxygen species via reduction by complex I of the respiratory chain at the inner matrix of the mitochondria [9], therefore increased mitochondrial fragmentation in MCEC cells were thought to be the result of increased of superoxide anion (O_2_^-^); lowering of the O_2_^-^ levels with an (O_2_^-^) inhibitor led to restoration of normal mitochondrial morphology [21]. Mitochondrial fragmentation has also been speculated to occur by free radical-induced solidification, or hardening, of the normally fluid and free flowing lipoprotein membrane followed by the shattering of the membrane resulting in fragmentation [22]. While this mechanism has been shown to exist in mitochondria in the presence of hydoxyl radicals [22], it is unclear whether the mitochondria in our experiment were fragmented in a similar manner.

Of interest was the finding that mitochondria in the 50 μM-treated lenses tended to contradict the general trend of increasing damage with increasing levels of menadione. At 216 h, the optical quality of the 50 μM concentration group was significantly better than that of the 1,000 μM group (p=0.0317, Figure 1), while no differences were detected between the 0 µM and the 1,000 µM groups. Moreover, the number of shorter mitochondria was greater in the 0 µM group compared to the 50 µM group (Figure 3), an indication of less damage to this latter group. The results suggest that low levels of menadione may actually be beneficial to both the optical quality of and mitochondrial integrity within the lens. The idea that toxins can have a hormesis effect, i.e., that a toxin that produces harmful effects at moderate and high doses can produce a positive effect at low doses [23], is not entirely new for mitochondria [24]. Previous findings by Shneyvays et al. [12] show that low concentrations of menadione can benefit mitochondria that have a defect in the electron transport chains, since the toxin can act as electron carriers. Although the mechanism behind the hormesis effect is unknown, and its existence even disputed, some believe that repair mechanisms activated due to the low levels of toxins also repair other damage that would have otherwise gone undetected [24,25].

In summary, results from the ScanTox™ system and confocal microscopy suggest that mitochondrial integrity and morphology are associated with optical quality of the lens. Further work into the effects of mitochondrial breakdown and cytoskeletal integrity would confirm the role of the cytoskeleton in optic function and cataract formation, which has been suggested for years [26].

## References

[1] Bantseev V Cullen AP Trevithick JR Sivak JG Optical function and mitochondrial metabolic properties in damage and recovery of bovine lens after in vitro carbonyl cyanide m-chlorophenylhydrazone treatment. Mitochondrion 2003 3 1 11 16120339 10.1016/S1567-7249(03)00059-X

[2] Bantseev V Sivak JG Confocal laser scanning microscopy imaging of dynamic TMRE movement in the mitochondria of epithelial and superficial cortical fiber cells of bovine lenses. Mol Vis 2005 11 518 23 16052167

[3] Trayhurn P Van Heyningen R The role of respiration in the energy metabolism of the bovine lens. Biochem J 1972 129 507 9 4643337 10.1042/bj1290507 PMC1174104

[4] Benedek GB Theory of transparency of eye. Appl Opt 1971 10 459 73 20094474 10.1364/AO.10.000459

[5] Bassnett S Mataic D Chromatin degradation in differentiating fiber cells of the eye lens. J Cell Biol 1997 137 37 49 9105035 10.1083/jcb.137.1.37 PMC2139849

[6] Bantseev VL Herbert KL Trevithick JR Sivak JG Mitochondria of rat lenses: distribution near and at the sutures. Curr Eye Res 1999 19 506 16 10550793 10.1076/ceyr.19.6.506.5279

[7] Bantseev V McCanna D Banh A Wong WW Moran KL Dixon DG Trevithick JR Sivak JG Mechanisms of ocular toxicity using the in vitro bovine lens and sodium dodecyl sulfate as a chemical model. Toxicol Sci 2003 73 98 107 12700424 10.1093/toxsci/kfg060

[8] Thor H Smith MT Hartzell P Bellomo G Jewell SA Orrenius S The metabolism of menadione (2-Methyl-1,4-Naphthoquinone) by isolated hepatocytes - a study of the implications of oxidative stress in intact-cells. J Biol Chem 1982 257 12419 25 6181068

[9] Iyanagi T Yamazaki I One-electron-transfer reactions in biochemical systems. 5. Difference in mechanism of quinone reduction by NADH dehydrogenase and NAD(P)H dehydrogenase (DT-diaphorase). Biochim Biophys Acta 1970 216 282 94 4396182 10.1016/0005-2728(70)90220-3

[10] De Marchi U Pietrangeli P Marcocci L Mondovi B Toninello A L-Deprenyl as an inhibitor of menadione-induced permeability transition in liver mitochondria. Biochem Pharmacol 2003 66 1749 54 14563485 10.1016/s0006-2952(03)00474-x

[11] Laux I Nel A Evidence that oxidative stress-induced apoptosis by menadione involves Fas-dependent and Fas-independent pathways. Clin Immunol 2001 101 335 44 11726226 10.1006/clim.2001.5129

[12] Shneyvays V Leshem D Shmist Y Zinman T Shainberg A Effects of menadione and its derivative on cultured cardiomyocytes with mitochondrial disorders. J Mol Cell Cardiol 2005 39 149 58 15893762 10.1016/j.yjmcc.2005.03.017

[13] Hegde KR Varma SD Combination of glycemic and oxidative stress in lens: implications in augmentation of cataract formation in diabetes. Free Radic Res 2005 39 513 7 16036327 10.1080/10715760400013755

[14] Wilson T Trends in confocal microscopy. Trends Neurosci 1989 12 486 93 2480661 10.1016/0166-2236(89)90104-5

[15] Wright SJ Centonze VE Stricker SA DeVries PJ Paddock SW Schatten G Introduction to confocal microscopy and three-dimensional reconstruction. Methods Cell Biol 1993 38 1 45 8246779 10.1016/s0091-679x(08)60998-x

[16] McCulley J, Stephens T. Draize eye testing alternatives-a perspective. New York: Mary Ann Liebert; 1993.

[17] Sivak JG Herbert KL Fonn D In vitro ocular irritancy measure of four contact lens solutions: damage and recovery. CLAO J 1995 21 169 74 7586475

[18] Sivak JG Yoshimura M Weerheim J Dovrat A Effect of hydrogen peroxide, DL-propranolol, and prednisone on bovine lens optical function in culture. Invest Ophthalmol Vis Sci 1990 31 954 63 2335456

[19] Legros F Lombes A Frachon P Rojo M Mitochondrial fusion in human cells is efficient, requires the inner membrane potential, and is mediated by mitofusins. Mol Biol Cell 2002 13 4343 54 12475957 10.1091/mbc.E02-06-0330 PMC138638

[20] Griparic L Kanazawa T van der Bliek AM Regulation of the mitochondrial dynamin-like protein Opa1 by proteolytic cleavage. J Cell Biol 2007 178 757 64 17709430 10.1083/jcb.200704112 PMC2064541

[21] Makino A Scott BT Dillmann WH Mitochondrial fragmentation and superoxide anion production in coronary endothelial cells from a mouse model of type 1 diabetes. Diabetologia 2010 53 1783 94 20461356 10.1007/s00125-010-1770-4 PMC2892085

[22] Dean RT Thomas SM Garner A Free-radical-mediated fragmentation of monoamine oxidase in the mitochondrial membrane. Roles for lipid radicals. Biochem J 1986 240 489 94 3814094 10.1042/bj2400489 PMC1147442

[23] Calabrese EJ Baldwin LA Defining hormesis. Hum Exp Toxicol 2002 21 91 7 12102503 10.1191/0960327102ht217oa

[24] Ristow M Zarse K How increased oxidative stress promotes longevity and metabolic health: The concept of mitochondrial hormesis (mitohormesis). Exp Gerontol 2010 45 410 8 20350594 10.1016/j.exger.2010.03.014

[25] Rattan SI Hormesis in aging. Ageing Res Rev 2008 7 63 78 17964227 10.1016/j.arr.2007.03.002

[26] Tagliavini J Gandolfi SA Maraini G Cytoskeleton abnormalities in human senile cataract. Curr Eye Res 1986 5 903 10 3802894 10.3109/02713688608995170

